# P-287. Real-World Sexually Transmitted Infection (STI) Testing Patterns Among Individuals Using Pre-Exposure Prophylaxis (PrEP) for HIV-1 Prevention in the United States

**DOI:** 10.1093/ofid/ofaf695.508

**Published:** 2026-01-11

**Authors:** Xiwen Huang, Juan Yang, Edward Gemson, Wenyi Wang, Li Tao, Dylan Mezzio, Chris Nguyen, Sandra I McCoy, Joshua Gruber

**Affiliations:** Gilead Sciences, Inc., Foster City, CA; Gilead Sciences, Inc. Foster City, CA, USA, Foster City, California; Gilead Sciences, Inc. Foster City, CA, USA, Foster City, California; Gilead Sciences, Inc. Foster City, CA, USA, Foster City, California; Gilead Sciences, Inc. Foster City, CA, USA, Foster City, California; Gilead Sciences Inc, Foster City, California; Gilead Sciences, Inc., Foster City, CA; Gilead Sciences, Inc. Foster City, CA, USA, Foster City, California; Gilead Sciences, Forest City, California

## Abstract

**Background:**

The Centers for Disease Control and Prevention recommends routine sexually transmitted infection (STI) testing every 3–6 months for people taking HIV-1 pre-exposure prophylaxis (PrEP), as part of comprehensive HIV-1/STI prevention services. However, real-world patterns of STI testing among individuals using PrEP in the US remain unclear.

**Table 1.**
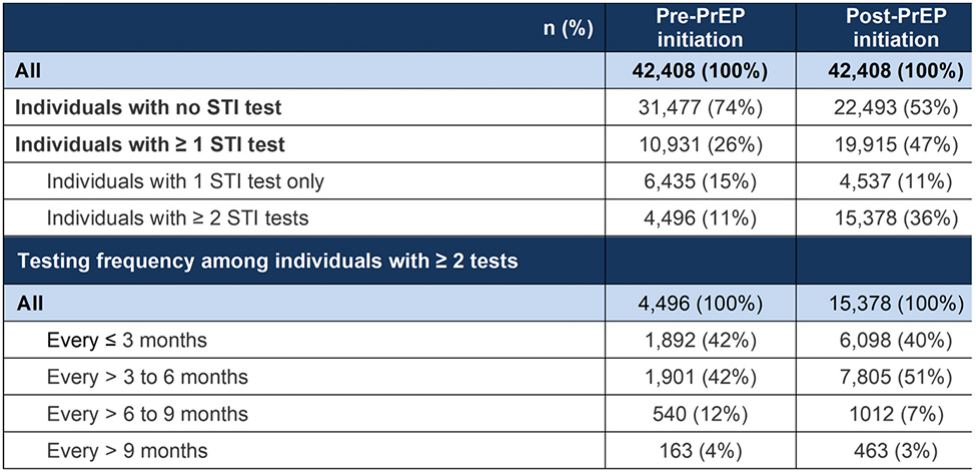
STI Testing in Adults Newly Prescribed PrEP Within the 12 Month Observation Window Pre- and Post-PrEP Initiation STI testing and prevalence were assessed 12 months pre- and post-PrEP initiation. STI tests conducted within 15 days of PrEP initiation (before or after) were counted in post-PrEP categories. PrEP, pre-exposure prophylaxis; STI, sexually transmitted infection.

**Figure 1.**
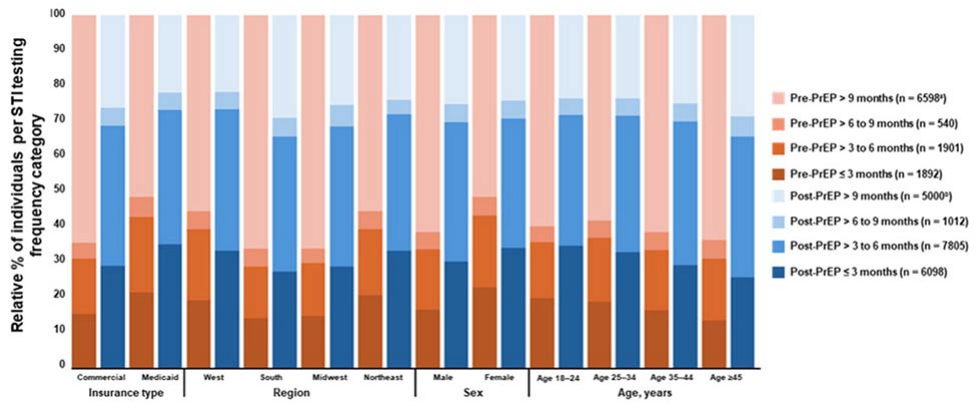
Frequency of STI Testing Among Individuals With ≥ 1 STI Test Within the 12 Month Observation Window Pre- and Post-PrEP Initiation STI tests conducted within 15 days of PrEP initiation (before or after) were counted in post-PrEP categories. a.Included individuals with one STI test only (n = 6435) and individuals with a testing frequency > 9 months (n = 163). b.Included individuals with one STI test only (n = 4537) and individuals with a testing frequency > 9 months (n = 463). PrEP, pre-exposure prophylaxis; STI, sexually transmitted infection.

**Methods:**

Adults (≥ 18 years) newly prescribed any PrEP regimen (October 2020 – December 2023) with ≥ 9 months cumulative use within the 12-months after PrEP initiation, were identified using HealthVerity. Testing and prevalence of chlamydia, gonorrhea, and syphilis were assessed 12 months pre- and post-PrEP initiation. Testing frequency was calculated as the average interval between ≥ 2 tests, categorized as every ≤ 3, > 3 to 6, > 6 to 9, and > 9 months. STI prevalence was defined as an STI diagnosis or positive laboratory result within each 12-month observation window.

**Figure 2.**
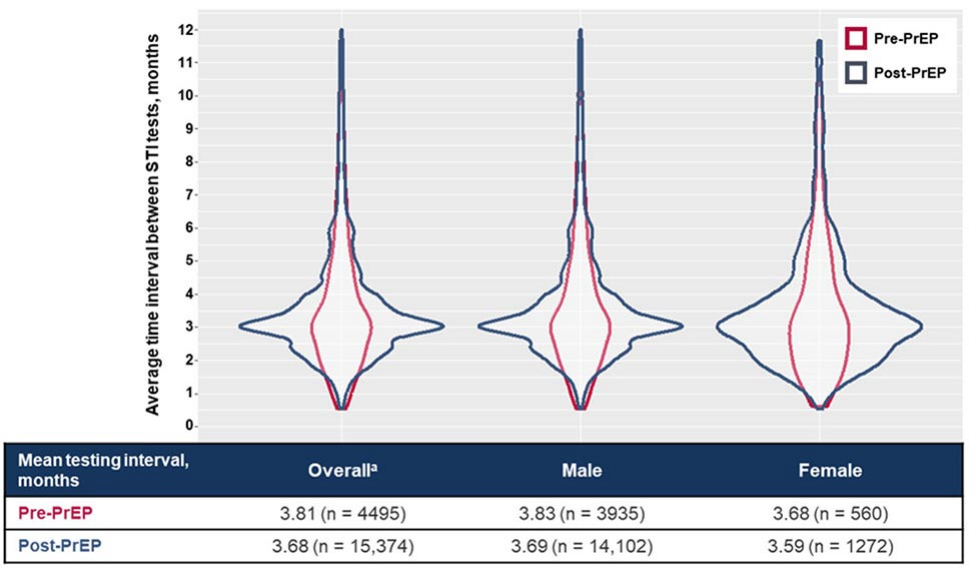
Mean STI Testing Intervals Among Individuals With ≥ 2 STI Tests in the 12 Month Pre- and Post-PrEP Initiation STI tests conducted within 15 days of PrEP initiation (before or after) were counted in post-PrEP categories. a.Individuals with unknown sex at birth were excluded. PrEP, pre-exposure prophylaxis; STI, sexually transmitted infection.

**Results:**

Among 42,408 PrEP-naive users, STI testing rates increased from 26% pre-PrEP to 47% post-PrEP initiation; 27% of all PrEP users tested within a 30-day window around PrEP initiation (± 15 days). While 33% of all PrEP users were tested every ≤ 6 months as recommended, 67% were tested every > 6 months or not at all. The proportion of individuals with ≥ 2 tests and the frequency of ≤ 6-monthly testing in these individuals increased post-PrEP use (Table 1). Individuals with commercial insurance, residing in the South or Midwest, or aged ≥ 45 years had the lowest pre-PrEP testing frequencies (Figure 1). After PrEP initiation, testing frequencies remained lowest in the South and among those aged ≥ 45 years, but increased in all other populations to approximately 30% testing every 3 months. Mean testing intervals decreased slightly from 3.8 to 3.7 months pre- and post-PrEP initiation, respectively (Figure 2). STI prevalence increased slightly from 12% pre-PrEP to 18% post-PrEP use, possibly due to increased testing.

**Conclusion:**

Overall, this study suggests that people using PrEP tested for STIs more frequently than before starting PrEP. However, only 33% of PrEP users tested every ≤ 6 months as recommended. As PrEP evolves with less frequent dosing options, further educational and structural interventions are needed to increase adherence with STI testing recommendations.

**Disclosures:**

Xiwen Huang, PhD, Gilead Sciences, Inc.: employee and shareholder Juan Yang, PhD, Gilead Sciences, Inc.: Employee and shareholder Edward Gemson, MS, Gilead Sciences, Inc.: employee and shareholder Wenyi Wang, MS, Gilead Sciences: Employee and shareholder Li Tao, PhD, Gilead Sciences, Inc.: Employee and shareholder Dylan Mezzio, PharmD, Gilead Sciences, Inc.: employee and shareholder Chris Nguyen, PharmD, Gilead Sciences, Inc.: Employee and shareholder Sandra I. McCoy, PhD, MPH, Gilead Sciences, Inc.: employee and shareholder Joshua Gruber, PhD MPH, Gilead Sciences: Employee and shareholder

